# *Pseudomonas aeruginosa* Isolates from Water Samples of the Gulf of Mexico Show Similar Virulence Properties but Different Antibiotic Susceptibility Profiles than Clinical Isolates

**DOI:** 10.1155/2024/6959403

**Published:** 2024-05-16

**Authors:** Luis E. Romero-González, Luis F. Montelongo-Martínez, Abigail González-Valdez, Sara E. Quiroz-Morales, Miguel Cocotl-Yañez, Rafael Franco-Cendejas, Gloria Soberón-Chávez, Liliana Pardo-López, Víctor H. Bustamante

**Affiliations:** ^1^Departamento de Microbiología Molecular, Instituto de Biotecnología, Universidad Nacional Autónoma de México, Cuernavaca, Morelos, Mexico; ^2^Departamento de Microbiología y Parasitología, Facultad de Medicina, Universidad Nacional Autónoma de México, Ciudad Universitaria, Ciudad de México, Coyoacán, Mexico; ^3^Departamento de Biología Molecular y Biotecnología, Instituto de Investigaciones Biomédicas, Universidad Nacional Autónoma de México, Ciudad Universitaria, Ciudad de México, Coyoacán, Mexico; ^4^Instituto Nacional de Rehabilitación “Luis Guillermo Ibarra Ibarra,” Ciudad de México, Mexico

## Abstract

*Pseudomonas aeruginosa* is an opportunistic pathogen found in a wide variety of environments, including soil, water, and habitats associated with animals, humans, and plants. From a One Health perspective, which recognizes the interconnectedness of human, animal, and environmental health, it is important to study the virulence characteristics and antibiotic susceptibility of environmental bacteria. In this study, we compared the virulence properties and the antibiotic resistance profiles of seven isolates collected from the Gulf of Mexico with those of seven clinical strains of *P. aeruginosa*. Our results indicate that the marine and clinical isolates tested exhibit similar virulence properties; they expressed different virulence factors and were able to kill *Galleria mellonella* larvae, an animal model commonly used to analyze the pathogenicity of many bacteria, including *P. aeruginosa*. In contrast, the clinical strains showed higher antibiotic resistance than the marine isolates. Consistently, the clinical strains exhibited a higher prevalence of class 1 integron, an indicator of anthropogenic impact, compared with the marine isolates. Thus, our results indicate that the *P. aeruginosa* marine strains analyzed in this study, isolated from the Gulf of Mexico, have similar virulence properties, but lower antibiotic resistance, than those from hospitals.

## 1. Introduction

*Pseudomonas aeruginosa* is a Gram-negative bacterium ubiquitously distributed in the environment. It is an opportunistic pathogen that exhibits high genomic plasticity and metabolic versatility, enabling its survival in a wide range of habitats including soil and different aquatic ecosystems such as wastewater, freshwater, and the sea [1, 2]. *P. aeruginosa* is a major cause of hospital-acquired infections, is recognized as one of the most life-threatening bacteria, and has been designated as a priority pathogen by the World Health Organization (WHO) for the research and development of new antibiotics [3, 4].

*P. aeruginosa* infections have been reported in various organisms, including plants, insects, fishes, mammals, and humans [5–8]. This bacterium has the ability to induce both acute and chronic infections in humans, including hospital-acquired pneumonia, cystic fibrosis-related respiratory infections, abdominal and urinary tract infections, and skin conditions such as folliculitis and external otitis [9]. Additionally, it is frequently associated with bacteremia, particularly in patients with severe burns or those with compromised immune system, such as patients with cancer or with acquired immune deficiency syndrome [10].

Numerous factors have been associated with the ability of *P. aeruginosa* to cause infections in hosts [11]. *P. aeruginosa* expresses various protein secretion systems, including a type three secretion system (T3SS), which is a syringe-like protein complex that translocates effector proteins/toxins from bacteria into the cytoplasm of host cells [12]. This T3SS secretes at least four effector proteins: ExoU, ExoS, ExoT, and ExoY; ExoS and ExoU are the most studied due to their clinical relevance [13]. ExoS-secreting strains induce cell apoptosis and actin cytoskeleton disruption [14], while ExoU secretion causes rapid cell integrity loss and lysis, promoting bacterial persistence and dissemination [15]. The production of ExoS and ExoU varies among *P. aeruginosa* strains and is mostly mutually exclusive, with strains expressing one of them, but not both [16]. Furthermore, the presence of these effectors correlates with the phylogenetic group of the *P. aeruginosa* strains; those from phylogroup 1 secrete ExoS, while those from phylogroup 2 secrete ExoU [17]. *P. aeruginosa* also expresses cell-associated components such as flagella, type IV pili, and lipopolysaccharide, as well as extracellular factors such as exopolysaccharides, rhamnolipids (RLs), pyocyanin, siderophores, and proteases [18, 19].

An additional virulence trait of *P. aeruginosa* is the ability to form biofilm. Biofilm formation depends on the production of a matrix of extracellular polymeric substances that maintain bacteria together [20]. Biofilm provides bacteria a survival strategy against temperature fluctuations, lack of nutrients, and the effect of antibiotics, thereby enhancing bacterial persistence on both living and nonliving surfaces [21, 22]. Additionally, quorum sensing (QS) is a pivotal factor in the virulence of *P. aeruginosa* [23]. QS is a bacterial regulatory system that coordinates the expression of multiple genes necessary for communal bacterial behavior; additionally, it controls virulence factors expression while promoting biofilm formation [24, 25].

The emergence of drug-resistant *P. aeruginosa* strains represents a global threat to human health. *P. aeruginosa* strains exhibit intrinsic resistance to several antibiotics, primarily due to the low permeability of the outer membrane and the expression of antibiotic efflux pumps [26]. Additionally, *P. aeruginosa* strains present acquired resistance to many antibiotics, which is mediated by the acquisition of genes encoding enzymes that inactivate antibiotics, as well as by mutations leading to overexpression of efflux pumps or to the modification of antibiotic target sites [3, 27].

*P. aeruginosa* strains present genomic plasticity, which reflects their adaptive nature [28]. However, a conservation of virulence-associated traits among clinical and environmental isolates of *P. aeruginosa* has been reported [29, 30]. A whole-genome comparison allows for the classification of *P. aeruginosa* strains into three major clades or groups [31]. Strains from group 1, including *P. aeruginosa* PAO1, are more common in both clinical and environmental niches than strains from groups 2 and 3, represented by *P. aeruginosa* PA14 and *P. aeruginosa* PA7, respectively [17, 32].

Most studies on the virulence and antibiotic resistance of *P. aeruginosa* have focused on clinical isolates. In the context of One Health, which emphasizes addressing health threats at the human-animal-environment interface, the study of environmental *P. aeruginosa* strains is essential [33]. Despite that *P. aeruginosa* strains have been isolated from the sea [34–38], there is limited information regarding their virulence and antibiotic resistance properties.

In this study, we analyzed different virulence phenotypes and the susceptibility to distinct antibiotics of *P. aeruginosa* strains isolated from the Gulf of Mexico (GoM), specifically comparing them to strains from hospital patients and clinical environments. As expected, we detected strains belonging to phylogroups 1 and 2 among the environmental and clinical isolates analyzed, and we found that the marine and clinical strains tested possess similar virulence traits. In contrast, the marine strains were more susceptible to antibiotics than the clinical strains, which correlates with the anthropogenic impact evaluated on these strains. Our study provides additional evidence showing the pathogenicity of environmental *P. aeruginosa* strains from the GoM. Furthermore, it illustrates the anthropogenic impact on the selection of antibiotic-resistant *P. aeruginosa* strains.

## 2. Materials and Methods

### 2.1. Bacterial Strains

Bacteria strains used in this study are listed in Table 1. *P. aeruginosa* marine isolates were previously recovered from seawater samples from the GoM and identified by the analysis of their 16S rRNA gene sequence [38, 39]. The *P. aeruginosa* isolates P1165, P1483, P1503, P1546, and P1547 were obtained from clinical samples in the Instituto Nacional de Rehabilitación (National Institute of Rehabilitation) Luis Guillermo Ibarra Ibarra in Mexico City and identified by a semiautomated system Vitek 2 Compact® (bioMereux Marcy l'Etoile, France). *P. aeruginosa* isolates P6103 and P3536 were obtained from clinical environments [40, 41]. Clinical isolates were confirmed as *P. aeruginosa* by phylogenetic analysis of their 16S rRNA gene using the EZBioCloud database [43] (Table 2). Additionally, all *P. aeruginosa* strains were phenotypically characterized by growth on cetrimide agar (Merck, Germany), a selective medium for where the production of pyocyanin and fluorescein pigments is observed.

### 2.2. Biofilm Formation

The biofilm-forming ability of *P. aeruginosa* strains was assessed using the crystal violet assay in polystyrene microtiter plates (Costar®, Corning Incorporated), as previously described [44]. Bacterial strains were cultured in lysogeny broth (LB) for 24 h at 37°C. Absorbance at 570 nm (OD_570_) was measured using a microtiter plate reader (BioTek™ Epoch2™, San Diego, CA, USA). Experiments were conducted in triplicate. A cut-off OD value (ODc) was calculated as three standard deviations (SDs) above the mean value of absorbance at OD_570_ of the negative control: ODc = mean value of OD5_570_ for negative control + (3 × SD of negative control). The final OD value (OD_*f*_) was calculated as the mean value of absorbance at OD_570_ of the respective strain minus the ODc value: OD_*f*_ = mean value of OD_570_–ODc value. Then, the bacterial strains were classified as previously described [44]. Strains with OD_*f*_ ≤ ODc were classified as no biofilm producer; ODc < OD_*f*_ ≤ 2 × ODc were classified as weak biofilm producers; 2 × ODc < OD_*f*_ ≤ 4 × ODc were classified as moderate biofilm producers, while OD_*f*_ > 4 × ODc were classified as strong biofilm producers.

### 2.3. Pyocyanin and Elastase Production

Pyocyanin and elastase production was assessed in *P. aeruginosa* cultures grown in LB. The elastase activity was measured using the previously described Elastin-Congo Red assay [37]. Pyocyanin production was quantified from bacterial culture supernatants as previously described [45]. The pyocyanin concentration in *µ*g ml^−1^ was calculated by multiplying the absorbance value at 520 nm with pyocyanin-specific molar extinction coefficient (17.072).

### 2.4. Antibiotic Susceptibility

The antibiotic susceptibility of *P. aeruginosa* strains was assessed using the microdilution method in cation-adjusted Mueller–Hinton broth, in accordance with the Clinical and Laboratory Standards Institute (CLSI) guidelines [46]. The tested antibiotics were ceftazidime (CAZ), piperacillin (PIP), meropenem (MER), norfloxacin (NOR), ciprofloxacin (CIP), gentamicin (GEN), imipenem (IMI), and amikacin (AMI). Multidrug resistance (MDR) was defined as nonsusceptibility to at least one antibiotic from three or more antibiotic categories, as described before [47]. *P. aeruginosa* ATCC 27853 was utilized as control strain for antimicrobial susceptibility testing. Antibiotics were purchased from Sigma-Aldrich.

### 2.5. Pathogenicity Assays

The virulence of *P. aeruginosa* isolates was analyzed using the *Galleria mellonella* (wax moth) infection model, following the established protocols [48]. Groups of ten *G. mellonella* larvae were inoculated with 10 µl of a bacterial suspension (1 × 10^2^ CFU in 1X PBS) through the lower left proleg using an insulin syringe. The infected larvae were then incubated at 30°C without food and monitored at different times postinjection to record mortality. *P. aeruginosa* PAO1, a human clinical isolate, and the nonpathogenic *Escherichia coli* DH5*α* strain were used as positive and negative controls, respectively.

### 2.6. Rhamnolipids Production

Bacterial strains were first cultured overnight in LB and then subcultured in a PPGAS medium with aeration at 37°C for 24 h [49]. Supernatants were obtained by centrifugation at 14,000 rpm for 10 min. Rhamnolipids were detected in the supernatants using thin-layer chromatography (TLC) following the method described by Matsuyama [50].

### 2.7. Detection of Secreted ExoS and ExoU Toxins

*P. aeruginosa* cultures grown in LB supplemented with 20 mM Mg_2_Cl + 5 mM EGTA, up to an OD_600_ 1.5, were used to detect ExoS and ExoU. Proteins from supernatants were precipitated with trichloroacetic acid and separated by 15% SDS-PAGE electrophoresis, transferred onto a nitrocellulose membrane, and probed by Western blot assays using anti-ExoS or anti-ExoU polyclonal antibodies, as reported previously [51]. Anti-GroEL polyclonal antibodies (Sigma) were used to detect the GroEL protein as a load control. Blots from membranes were developed with the HRP luminol Super Signal chemiluminescent substrate (Thermo Scientific).

### 2.8. Class 1 Integron Identification

Detection of the *intI1* gene was performed as previously described [52]. Purified genomic DNA was used as the template to amplify by PCR a fragment of *intI1* using the HS463a and HS464 primers (Table 3). PCRs were performed using GoTaq Flexi DNA Polymerase (Promega, Wisconsin, USA) according to the manufacturer's instructions. Amplification conditions included an initial denaturation at 94°C for 5 min, followed by 35 cycles of 30 s denaturation at 94°C, 30 s annealing at 60°C, 90 s extension at 72°C, and a final extension step at 72°C for 5 min.

### 2.9. Phylogroup Identification

PCR amplification of *exoS* and *PA14300* genes was used to identify *P. aeruginosa* phylogroups as proposed before [53]. PCRs were performed using GoTaq Flexi DNA Polymerase (Promega, Wisconsin, USA). Briefly, a 25 *µ*l PCR mixture was prepared, containing 2 *µ*l of purified genomic DNA, 5 *µ*l of 5X Green GoTaq buffer, 1 *µ*l of 4% DMSO, 2.5 *µ*l of 25 mM MgCl2, 2 *µ*l each oligonucleotide (1.0 *µ*M), 0.4 *µ*l of GoTaq enzyme (1.25 U), 1 *µ*l of a dNTPs mix (10 *µ*M each), and sterile water to reach the final volume. Amplification conditions included an initial denaturation step at 95°C for 3 min, followed by 30 cycles of denaturation at 95°C for 30 s, annealing at 59°C for 30 s, and elongation at 72°C for 75 s, with a final elongation step at 72°C for 5 min. The oligonucleotides used are shown in Table 3.

### 2.10. Statistical Analysis

Data are shown as mean ± standard deviation. Statistical analysis was carried out by using GraphPad Prism version 8.0.1 (GraphPad Software, San Diego, CA, USA), with one-way ANOVA combined with Dunnett's multiple comparison test, or the log-rank test, as required. A *P* value of <0.05 was considered statistically significant.

## 3. Results

### 3.1. The Marine and Clinical *P. aeruginosa* Isolates Tested Show Similar Virulence in *G. mellonella*

**Figure 1:**
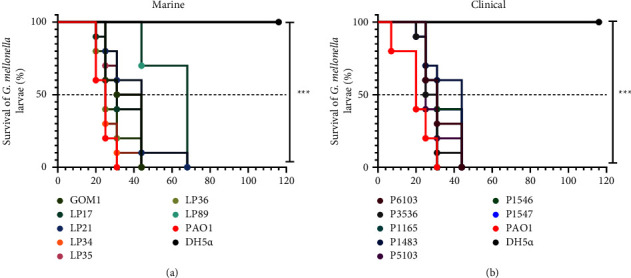
Marine and clinical *P. aeruginosa* isolates show similar virulence in *G. mellonella*. *G. mellonella* larvae were infected with 1 × 10^2^ CFU of marine or clinical *P. aeruginosa* strains, and their survival was monitored for 5 days. Kaplan–Meier survival curves of infected larvae with marine (a) or clinical (b) strains were generated. Control groups included *P. aeruginosa* PAO1 (pathogenic) and *E. coli* DH5*α* (nonpathogenic). Larvae not injected or injected with 1X PBS did not kill any larvae (data not shown). *P* values were calculated using the log-rank test. ^*∗∗∗*^, *P* < 0.001.

The *G. mellonella* larvae are highly susceptible to *P. aeruginosa* infection, making them a suitable model for assessing the pathogenicity of this bacterium [54]. Thus, we evaluated the virulence of seven *P. aeruginosa* isolates from the GoM and, for comparison, seven clinical isolates of *P. aeruginosa* in *G. mellonella* larvae. All clinical isolates and five out of the seven marine isolates caused death of the 100% of the larvae at 44 h postinfection, a phenotype similar to that of the *P. aeruginosa* PAO1 strain used as a positive virulence control (Figures 1(a) and 1(b)). The virulence of the *P. aeruginosa* PAO1 strain has been previously demonstrated in *G. mellonella* and mice [55, 56]. The other two marine strains killed 100% of the larvae at 68 h postinfection (Figure 1(a)). As expected, injection with the nonvirulent strain *E. coli* DH5*α* did not induce larval mortality (Figure 1). These results indicate that, in overall terms, the marine and clinical isolates of *P. aeruginosa* tested show similar virulence in the *G. mellonella* larvae.

### 3.2. The Marine and Clinical *P. aeruginosa* Isolates Tested Form Biofilm

**Figure 2:**
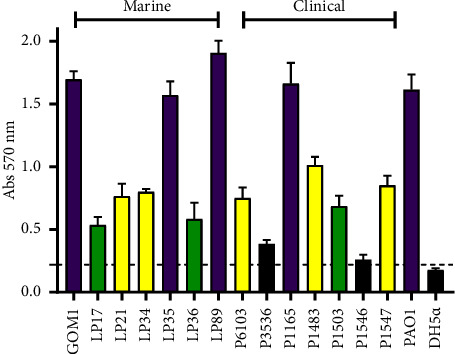
Marine and clinical *P. aeruginosa* isolates form biofilm. Biofilm formation was quantified using the crystal violet staining method. The strains were classified based on their biofilm-producing capacity: strong (purple), moderate (yellow), weak (green), or not biofilm forming (black), following the criteria established by Stepanović [44]. The *P. aeruginosa* PAO1 and *E. coli* DH5*α* strains were used as positive and negative controls of biofilm formation, respectively. The dotted line shows the cut-off value used for the classification of biofilm formation. The bars represent the average of three independent experiments ± SD.

Biofilm formation has been reported as an important factor contributing to the virulence of *P. aeruginosa* [57]. We compared the capacity of the marine and clinical *P. aeruginosa* isolates to form biofilm in plastic microplates. Both the marine and the clinical isolates exhibited different levels of biofilm formation (strong, moderate, or weak); the seven marine isolates formed biofilm, whereas two out of the seven clinical isolates did not form biofilm (Figure 2). The *P. aeruginosa* PAO1 and *E. coli* DH5*α* strains, used as positive and negative controls of biofilm formation, respectively, presented the expected phenotype (Figure 2). These results show that the tested marine isolates can form biofilm like clinical isolates.

### 3.3. The Marine and Clinical *P. aeruginosa* Isolates Tested Express Factors Associated with Virulence

**Figure 3:**
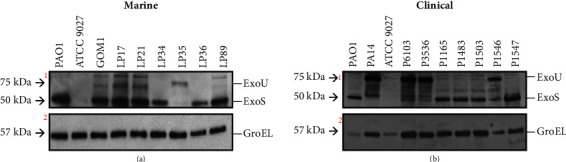
Detection of ExoS and ExoU toxins produced by *P. aeruginosa* isolates. The presence of ExoS or ExoU toxins in the culture supernatant of the marine (a) and clinical (b) *P. aeruginosa* strains was assessed by the Western blot using anti-ExoS and anti-ExoU polyclonal antibodies. As a load control, GroEL was detected by using anti-GroEL polyclonal antibodies. Positive controls for ExoS and ExoU were the *P. aeruginosa* PAO1 and PA14 strains, respectively, whereas the negative control was the *P. aeruginosa* ATCC9027 strain, which does not produce any of these toxins. Blots marked with numbers 1 and 2 were obtained with a mix of the anti-ExoS and anti-ExoU antibodies, and only with anti-GroEL antibodies, respectively.

Several factors have been associated with the virulence of *P. aeruginosa*, including the ExoS and ExoU toxins, pyocyanin, elastase, and rhamnolipids [58, 59]. We determined the production of these factors in the marine and clinical *P. aeruginosa* strains assessed in our study. *P. aeruginosa* secretes the toxins ExoS and ExoU through a T3SS; ExoS is a dual function protein with GTPase-activating proteins and ADP-ribosyltransferase domains that disrupt the host actin cytoskeleton and impair cell-to-cell adhesion, whereas ExoU, found in cytotoxic *P. aeruginosa* strains, has a phospholipase activity and significantly contributes to infection severity [16, 60]. To evaluate the expression and secretion of ExoS and ExoU, we detected the presence of these proteins in the supernatant of bacterial cultures. Our findings demonstrated that six out of the seven marine isolates and four out of the seven clinical isolates secreted ExoS, a phenotype displayed by the *P. aeruginosa* PAO1 strain (Figure 3). Meanwhile, one marine strain and three clinical isolates secretes ExoU, a phenotype displayed by the *P. aeruginosa* PA14 strain (Figure 3). As expected, ExoS and ExoU toxins were not detected in the *P. aeruginosa* ATCC 9027 strain, which was included as a negative control (Figure 3). These results demonstrate that the marine and clinical isolates assessed can produce and secrete ExoS or ExoU toxins.

**Figure 4:**
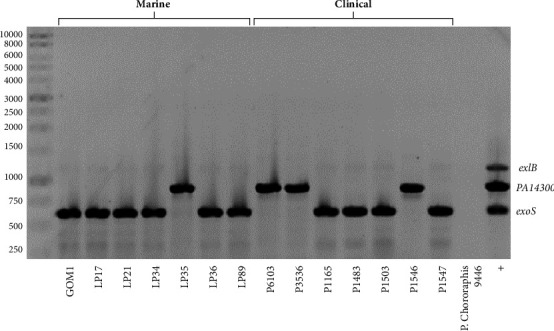
Identification of phylogroups in marine and clinical *P. aeruginosa* isolates. Amplification by PCR of *exoS* or *PA14300* genes in *P. aeruginosa* isolates. Strains from group 1 amplify a 621-bp fragment corresponding to the *exoS* gene, strains from group 2 amplify an 888-bp fragment corresponding to the *PA14300* gene. *P. chlororaphis* 9446 strain was used as a negative control in these assays. PCR products for *exoS* (group 1), *PA14300* (group 2), and *exlB* (group 3) genes are shown (lane +).

The genomic analysis of *P. aeruginosa* has revealed the presence of two main groups within its population structure [31]. These groups exhibit distinct production patterns of the effector proteins ExoS and ExoU [17]. To determine the phylogroup for the marine and clinical *P. aeruginosa* isolates tested in our study, we conducted a PCR analysis focused on the presence of the *exoS* and *PA14300* genes, which have been found to be significant in delineating the major groups 1 and 2 of *P. aeruginosa*, respectively [53]. The *exoS* gene was detected in six marine isolates and four clinical isolates, while the *PA14300* gene was detected in one marine isolate and three clinical isolates (Figure 4). As expected, the detection of the *exoS* or *PA14300* genes by PCR correlated with the detection of the ExoS or ExoU proteins by the Western blot (Figures 3 and 4). No amplification of the *exoS* or *PA14300* genes was obtained for the *P. chlororaphis* 9446 strain that was used as a negative control (Figure 4). Together, our analysis supports that, among the *P. aeruginosa* isolates tested, six marine and four clinical isolates belong to group 1, whereas one marine and three clinical isolates belong to group 2. These results are in agreement with previous studies showing the presence of phylogroups 1 and 2 among clinical and environmental isolates [17, 32].

**Figure 5:**
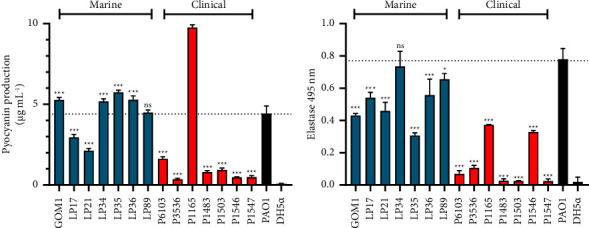
Pyocyanin and elastase production by marine and clinical *P. aeruginosa* isolates. Pyocyanin (a) and elastase (b) production was determined in the marine (blue bars) and clinical (red bars) *P. aeruginosa* isolates assessed. The *P. aeruginosa* PAO1 and *E. coli* DH5*α* strains (both indicated by black bars) were used as positive and negative controls, respectively. The bars represent the average of three independent experiments ± SD. *P* value was calculated using one-way ANOVA combined with Dunnett's test for comparison of the isolates with the *P. aeruginosa* PAO1 strain. ^*∗∗∗*^, *P* < 0.001. ns: nonsignificant; ^∗^, *P* < 0.05.

Pyocyanin and elastase are additional virulence-associated factors synthesized by *P. aeruginosa* [61]. Pyocyanin, a phenazine molecule, is associated with the generation of reactive oxygen species, while elastase is a protease that facilitates bacterial colonization and leads to tissue damage in hosts [62, 63]. The production of these factors was detected and compared between the analyzed marine and clinical *P. aeruginosa* isolates. The *P. aeruginosa* PAO1 and *E. coli* DH5*α* strains were assessed as positive and negative controls, respectively. The marine and clinical isolates exhibited different levels of pyocyanin production; in general terms, the marine isolates showed higher levels of pyocyanin than the clinical isolates (Figure 5(a)). Five out of the seven marine isolates presented pyocyanin levels equal to or higher than the *P. aeruginosa* PAO1 strain, while only one clinical isolate showed pyocyanin levels higher than those shown by the *P. aeruginosa* PAO1; this last clinical isolate exhibited the highest pyocyanin production among all strains tested (Figure 5(a)). On the other hand, the marine isolates showed a higher elastase production than most of the clinical isolates; except for one marine isolate, the rest of the isolates tested presented lower levels of elastase than the *P. aeruginosa* PAO1 strain (Figure 5(b)). As expected, pyocyanin or elastase production was not observed in the *E. coli* DH5*α* strain (Figures 5(a) and 5(b)). Collectively, these results indicate that the analyzed marine isolates exhibit pyocyanin and elastase production levels comparable to or even higher than those shown by clinical isolates.

**Figure 6:**
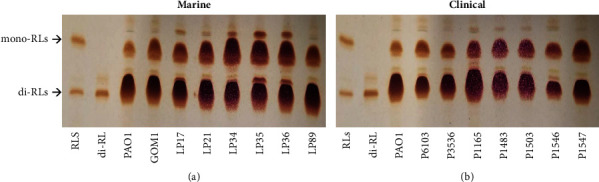
Rhamnolipid production by marine and clinical *P. aeruginosa* isolates. Production of RLs was determined by TLC in the culture supernatants of the marine (a) or clinical (b) *P. aeruginosa* isolates. As controls, a mixture of mono-RL and di-RLs, only di-RLs, and the *P. aeruginosa* PAO1 strain (produces both types of RLs) were assessed.

Extracellular virulence factors, including RLs, play a role in the virulence of *P. aeruginosa* [64]. RLs are amphiphilic glycolipids with detergent and solubilizing properties; *P. aeruginosa* produces both mono-RLs (containing 1 molecule of rhamnose) and di-RLs (containing 2 molecules of rhamnose) [65]. RL production was detected by TLC in the culture supernatants of the marine and clinical *P. aeruginosa* isolates studied. Purified mono- and di-RLs, and the *P. aeruginosa* PAO1 strain that produces both RL types, were assessed as positive controls. All the marine and clinical isolates, and the *P. aeruginosa* PAO1 strain, produced both mono- and di-RLs (Figure 6). These results indicate that the marine and clinical isolates tested produce mono- and di-RLs like the *P. aeruginosa* PAO1 strain.

### 3.4. The Marine Isolates Tested Show Low Antibiotic Resistance Compared to Clinical Isolates

**Figure 7:**
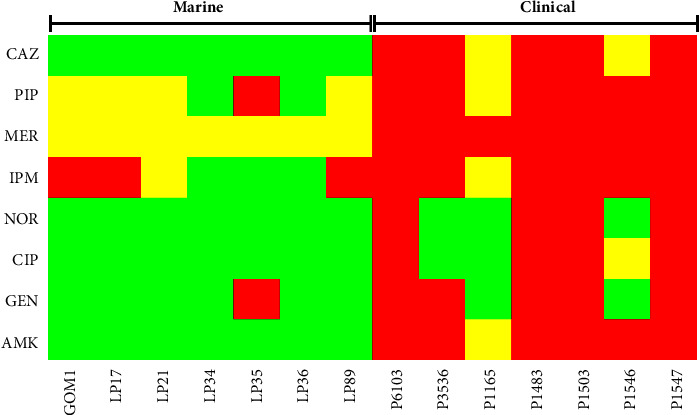
Antibiotic susceptibility of marine and clinical *P. aeruginosa* isolates. Heatmap of antibiotic susceptibility to selected antibiotics. Strains were classified as resistant (red), intermediate resistant (yellow), or susceptible (green) by determining the minimum inhibitory concentration (MIC) as indicated by CLSI guidelines. All tests were performed in triplicate. The tested antibiotics were ceftazidime (CAZ), piperacillin (PIP), meropenem (MER), norfloxacin (NOR), ciprofloxacin (CIP), gentamicin (GEN), imipenem (IMI), and amikacin (AMI).

Antibiotic resistance is a widespread feature in clinical bacteria, but it can also be present in environmental bacteria [66]. We analyzed the susceptibility of the marine and clinical isolates studied to antibiotics commonly used to treat *P. aeruginosa* infections (ceftazidime, piperacillin, meropenem, imipenem, norfloxacin, ciprofloxacin, gentamicin, and amikacin). As could be expected, most of the clinical isolates displayed resistance or intermediate resistance to most of the antibiotics tested; the P1165 isolate presented the profile with the lowest level of antibiotic resistance among the clinical isolates (Figure 7). In contrast, the marine isolates showed susceptibility or intermediate resistance to most of the antibiotics tested; the LP35 isolate presented the profile with the highest level of antibiotic resistance among the marine isolates (Figure 7). These results indicate that the marine isolates studied have low antibiotic resistance compared to clinical isolates; interestingly, three marine isolates presented resistance to piperacillin, meropenem, or imipenem.

### 3.5. Anthropogenic Impact on Marine and Clinical *P. aeruginosa* Isolates Tested

**Figure 8:**
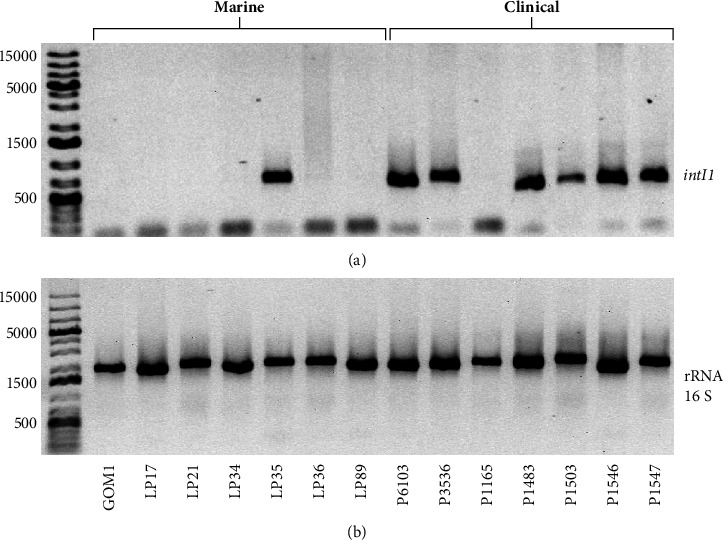
Detection of the *intI1* gene in marine and clinical *P. aeruginosa* isolates. Agarose gel electrophoresis of the PCR products of the *intI1* gene (a) and the rRNA 16S gene used as a positive control (b). The same results were obtained in three different assays.

The impact of human activities on bacteria can be evaluated by the presence of class 1 integrons; these genetic elements mediate the recruitment and mobilization of antibiotic resistance genes, their abundance can change rapidly in response to selection pressure from pollutants such as biocides and antibiotics, and they can be found in both pathogenic and nonpathogenic bacteria [67, 68]. To analyze the anthropogenic impact on the marine and clinical *P. aeruginosa* isolates studied, we detected the *intI1* gene by PCR, which encodes the integrase of class 1 integrons [52, 69]. The *intI1* gene was found in one out of the seven marine isolates and in six out of the seven clinical isolates (Figure 8). As could be expected, these results reveal a higher anthropogenic impact on the clinical isolates than on the marine isolates tested. Interestingly, the marine isolate positive for *intI1* (LP35) had the highest antibiotic resistance profile among the marine isolates; conversely, the clinical isolate negative for *intI1* (P1165) had the lowest antibiotic resistance profile among the clinical isolates (Figures 7 and 8).

## 4. Discussion

*P. aeruginosa* is an opportunist pathogen ubiquitously distributed; thus, it is important to know if its presence in the environment can represent a risk for human health. In this study, we found that all tested *P. aeruginosa* isolates from the GoM exhibit virulence properties similar to those observed in clinical isolates.

We found that both the marine and clinical isolates of *P. aeruginosa* tested can kill *G. mellonella* larvae at similar infection doses and mortality rates. Previous studies have also shown that environmental strains of *P. aeruginosa* are virulent in the *G. mellonella* model [70, 71]. In addition, our results showed that the marine and clinical isolates of *P. aeruginosa* tested form biofilm at different levels. Biofilm serves as a resistance mechanism against environmental challenges, including antibiotics, and is an important virulence factor for tissue and medical device colonization [72, 73]. Biofilm is the major mode of bacteria life in the environment, including the marine habitats, enabling the formation of complex microbial communities [74, 75]. Several studies have reported that marine strains of *P. aeruginosa* are capable of forming biofilm [76, 77]. Furthermore, our results indicate that the marine and clinical isolates of *P. aeruginosa* tested express factors related to the virulence of this bacterium (secretion of ExoS and ExoU toxins through the T3SS, as well as production of pyocyanin, elastase, and rhamnolipids). Even, the marine isolates tested produced higher levels of pyocyanin and elastase than the clinical isolates, supporting that these factors not only contribute to virulence but also have an ecological function, enabling the bacteria to thrive in diverse environments [78].

Of the seven marine isolates tested, six secreted ExoS toxin and only one isolate secreted ExoU, while of the seven clinical isolates tested, four secreted ExoS and three secreted ExoU. Consistent with these results, it has been reported that ExoS is more prevalent than ExoU in both environmental and clinical isolates of *P. aeruginosa* [79]. The secretion of the ExoS and ExoU effectors has a phylogenetic significance since the phylogroup 1 strains produce ExoS, while those of phylogroup 2 ExoU [17]. Our results also confirm these findings.

The secretion of toxins such as ExoS, ExoT, ExoU, and ExoY, through the T3SS, has been associated with increased virulence in *P. aeruginosa* [80]. The secretion of ExoS and ExoU toxins further indicates that both the marine and clinical *P. aeruginosa* isolates tested have a functional T3SS. The T3SSs are associated with virulence but are also found in nonpathogenic bacteria [81]. A previous genomic analysis identified T3SS genes in 109 different bacterial genera, including environmental bacteria unrelated to eukaryotic hosts, beneficial plant bacteria like rhizobia, and some *Pseudomonas* isolates [82].

As described above, the 14 marine and clinical *P. aeruginosa* isolates tested in our study have a functional T3SS. In agreement with our results, a previous whole-genome analysis showed a very low prevalence (1.5%) of *P. aeruginosa* strains lacking the T3SS and producing Exolysin A; interestingly, these PA7-like strains present a moderate virulence [83].

Different studies have reported the production of pyocyanin and elastase in environmental strains of *P. aeruginosa* [37, 84, 85]. Pyocyanin is a redox-active pigment that acts as an electron acceptor and has ecological functions related to signaling [86]. Elastase B is the predominant protease in the *P. aeruginosa* secretome, and its ecological function is related to nutrient acquisition through protein degradation [87]. On the other hand, the production of RLs has also been reported in marine strains of *P. aeruginosa* [37, 88–90] and contributes to various biofilm-related processes such as formation, maturation, dispersal, and bacterial propagation within the biofilm [91, 92].

In summary, our findings support previous research, indicating that all *P. aeruginosa* strains possess the potential to be pathogenic regardless of their ecological habitats, including marine environments [30].

In addition to their virulence properties, we also compared the antibiotic susceptibility of the analyzed marine and clinical isolates of *P. aeruginosa*. Evolution of antibiotic resistance is a complex process influenced by numerous factors. The primary driver of resistant bacteria is the selective pressure from human antibiotic usage, particularly in clinical environments [93]. The marine *P. aeruginosa* isolates we tested were more susceptible to antibiotics than clinical isolates, which is consistent with previous reports, indicating a lower prevalence of antibiotic resistance in environmental isolates than in clinical isolates of *P. aeruginosa* [94, 95]. However, some of the marine *P. aeruginosa* isolates we analyzed showed resistance to carbapenems (meropenem or imipenem). Previous studies indicate that the increased efflux system activity contributes to antibiotic resistance of environmental *P. aeruginosa* strains, including those from aquatic environments [96, 97]. Furthermore, previous research has identified genetic factors for antibiotic resistance in bacteria from pristine natural environments such as caves, deep water, and subsurface regions [98–101].

Antibiotic resistance arises mainly from antibiotic exposure caused by human activities. Class 1 integrons contribute to the acquisition and dissemination of antibiotic resistance genes and serve as markers to evaluate the anthropogenic impact in bacteria [67, 102]. Numerous studies have demonstrated a strong correlation between the prevalence of class 1 integrase *intI1* and the degree of anthropogenic impact and pollution in various environments [68, 103, 104]. We determined the presence of the class 1 integron in the isolates of *P. aeruginosa* in the study. As could be expected, the *intI1* gene was found in nearly all the clinical isolates (6 out of 7) and in only one of the seven marine isolates, indicating a greater anthropogenic influence in the clinical isolates than in the marine isolates tested. Notably, the marine isolate with the lowest antibiotic susceptibility, LP35, possessed the class 1 integron. The aquatic environment is recognized as a major reservoir and dissemination route for antibiotic resistance worldwide [105]. Several studies have documented the presence of class 1 integrons in antibiotic-resistant *P. aeruginosa* strains from various environments, including aquatic environments [106, 107]. Consistently, a previous study found a higher prevalence of class 1 integrons in clinical *P. aeruginosa* compared to environmental isolates [108].

## 5. Conclusions

Our study, together with previous reports, provides evidence supporting that *P. aeruginosa* marine isolates possess similar virulence properties but less antibiotic resistance than clinical strains. Additionally, as could be expected, our results show a lower presence of class 1 integrons, a marker of anthropogenic impact, in the *P. aeruginosa* marine isolates tested than in clinical strains.

## Figures and Tables

**Table 1:** Bacterial strains used in this study.

Strains	Relevant features^a^	Reference or source
*P. aeruginosa* GOM1	Isolated from seawater (55 m depth)	[38]
*P. aeruginosa* LP17	Isolated from seawater (55 m depth)	[39]
*P. aeruginosa* LP21	Isolated from seawater (55 m depth)	[39]
*P. aeruginosa* LP34	Isolated from seawater (1000 m depth)	[39]
*P. aeruginosa* LP35	Isolated from seawater (1000 m depth)	[39]
*P. aeruginosa* LP36	Isolated from seawater (1000 m depth)	[39]
*P. aeruginosa* LP89	Isolated from seawater (30 m depth)	[39]
*P. aeruginosa* P6103	Nosocomial isolate	[40]
*P. aeruginosa* P3536	Environmental hospital isolate	[41]
*P. aeruginosa* P1165	Isolated from blood culture	This study
*P. aeruginosa* P1483	Isolated from endotracheal aspirate	This study
*P. aeruginosa* P1503	Isolated from tendon tissue	This study
*P. aeruginosa* P1546	Isolated from infected wound	This study
*P. aeruginosa* P1547	Isolated from urine culture	This study
*P. aeruginosa* PAO1 (ATCC15692)	Reference strain	American-type culture collection (ATCC)
*P. aeruginosa* 27853	Reference strain	American-type culture collection (ATCC)
*P. aeruginosa* 9027	Reference strain	American-type culture collection (ATCC)
*P. aeruginosa* PA14	Reference strain	[42]
*Escherichia coli* 25922	Reference strain	American-type culture collection (ATCC)
*Escherichia coli* DH5*α*	Laboratory strain	Invitrogen

^a^Marine strains from the same depth were isolated from different water samples taken in the same station of the GoM.

**Table 2:** Identification of *P. aeruginosa* isolates by 16S rRNA gene sequence analysis.

Isolate	Bacteria with best match of 16S rRNA gene^a^	Query cover (%)	Identity (%)
P6103	*P. aeruginosa* strain JCM 5962 (BAMA01000316.1)	98	99.37
P3536	*P. aeruginosa* strain JCM 5962 (BAMA01000316.1)	98	99.87
P1165	*P. aeruginosa* strain JCM 5962 (BAMA01000316.1)	98	99.93
P1483	*P. aeruginosa* strain JCM 5962 (BAMA01000316.1)	98	99.65
P1503	*P. aeruginosa* strain JCM 5962 (BAMA01000316.1)	98	99.01
P1546	*P. aeruginosa* strain JCM 5962 (BAMA01000316.1)	97	99.29
P1547	*P. aeruginosa* strain JCM 5962 (BAMA01000316.1)	98	99.16

^a^Accesion number is indicated between parentheses.

**Table 3:** Primers used in this work.

Primers	Sequence (5′ to 3′)	Target gene	Reference or source
*For class 1 integron identification*			
HS463a	CTGGATTTCGATCACGGCACG	*intI1*	[52]
HS464	ACATGCGTGTAAATCATCGTCG	*intI1*	[52]

*For phylogroup classification*			
exoS-Fwd	CAATCGCTTCAGCAGAGTC	*exoS*	This work
*exoS*-Rv	CAACTGGTCGGTGATTTCG	*exoS*	This work
PA14300-Fwd	GCGTTGATACTCAAGGCGTTTG	*PA14300*	This work
PA14300-Rv	GAGGGGGATGTCGGCAAG	*PA14300*	This work

## Data Availability

All data supporting the findings of this study are available within the paper.
